# Matrix metalloproteinase 9 a potential major player connecting atherosclerosis and osteoporosis in high fat diet fed rats

**DOI:** 10.1371/journal.pone.0244650

**Published:** 2021-02-11

**Authors:** Maha Sabry, Seham Mostafa, Laila Rashed, Marwa Abdelgwad, Samaa Kamar, Suzanne Estaphan

**Affiliations:** 1 Physiology Department, Faculty of Medicine, Cairo University, Giza, Egypt; 2 Medical Biochemistry and Molecular Biology Department, Faculty of Medicine, Cairo University, Giza, Egypt; 3 Histology Department, Faculty of Medicine, Cairo University, Giza, Egypt; 4 ANU Medical School, Australian National University, Canberra, ACT, Australia; Universite de Nantes, FRANCE

## Abstract

**Background:**

Cardiovascular diseases (CVD) represent one of the major sequelae of obesity. On the other hand, the relationship between bone diseases and obesity remains unclear. An increasing number of biological and epidemiological studies suggest the presence of a link between atherosclerosis and osteoporosis, however, the precise molecular pathways underlying this close association remain poorly understood. The present work thus aimed to study Matrix Metalloproteinase 9 (MMP-9), as a proposed link between atherosclerosis and osteoporosis in high fat diet fed rats.

**Methods and findings:**

40 rats were randomly divided into 4 groups: control, untreated atherosclerosis group, atherosclerotic rats treated with carvedilol (10mg/kg/d) and atherosclerotic rats treated with alendronate sodium (10mg/kg/d). After 8 weeks, blood samples were collected for estimation of Lipid profile (Total cholesterol, HDL, TGs), inflammatory markers (IL-6, TNF-α, CRP and NO) and Bone turnover markers (BTMs) (Alkaline phosphatase, osteocalcin and pyridinoline). Rats were then euthanized and the aortas and tibias were dissected for histological examination and estimation of MMP-9, N-terminal propeptide of type I procollagen (PINP), C-terminal telopeptide of type I collagen (CTX) and NF-kB expression. Induction of atherosclerosis via high fat diet and chronic stress induced a significant increase in BTMs, inflammatory markers and resulted in a state of dyslipidaemia. MMP-9 has also shown to be significantly increased in the untreated atherosclerosis rats and showed a significant correlation with all measured parameters. Interestingly, Carvedilol and bisphosphonate had almost equal effects restoring the measured parameters back to normal, partially or completely.

**Conclusion:**

MMP-9 is a pivotal molecule that impact the atherogenic environment of the vessel wall. A strong cross talk exists between MMP-9, cytokine production and macrophage function. It also plays an important regulatory role in osteoclastogenesis. So, it may be a key molecule in charge for coupling CVD and bone diseases in high fat diet fed rats. Therefore, we suggest MMP-9 as a worthy molecule to be targeted pharmacologically in order to control both conditions simultaneously. Further studies are needed to support, to invest and to translate this hypothesis into clinical studies and guidelines.

## Introduction

Obesity is a major risk factor for CVD. Even in the absence of other risk factors, obesity has been considered a strong independent predictor of CVD [1].

On the other hand, obesity has been thought to be protective against osteoporosis. Yet, in fact, The recent clinical and epidemiologic reports have illustrated a higher incidence of osteoporosis and bone fractures in obese individuals than in individuals within the normal weight range [2–4]. The complex relationship between obesity and osteoporosis is currently unclear.

Mounting evidence demonstrate that CVD patients are more prone to bone fractures, and that those with low bone mass exhibit a higher mortality caused by cardiovascular emergencies [5, 6]. In effect, some common anatomical and pathophysiological features interestingly govern both vascular calcification and bone mineralization. Arterial calcification is not a passive process of minerals precipitation, it is a well systematized process controlled by factors chiefly described in the osteogenesis process such as the ALP (alkaline phosphatase) and the OPN (osteopontin) [7].

Besides, common pathophysiological mechanisms, including inflammatory cytokines, dyslipidaemia, endogenous sex hormones, oxidized lipids, and oxidative stress, are involved in the progression of both conditions [8].

Currently, there is a strong interest in identifying a pivotal link tying atherosclerosis and osteoporosis, in order to search for therapeutic strategies that target the cardiovascular and the skeletal systems simultaneously.

Recent attention has been focused on extracellular matrix (ECM) and matrix metalloproteinase (MMP) as a novel disease marker as well as a therapeutic target to control tissue remodelling and disease progression.

Despite the number of studies that demonstrate increased level of expression of MMP-9 in atherosclerotic lesions [9] as well as in osteoclastogenesis [10], few studies have been designed to reveal the contributory role of MMP-9 in atherosclerosis, or to invest the possibility that MMP-9 may be a key molecule in charge for coupling these two pathologies [11].

### Aim of the work

The present work thus aimed to study:

The possible association between atherosclerosis and osteoporosis pathogenesis in high fat diet (HFD) fed rats through investigating the effect of induction of atherosclerosis on bone turnover markers (BTMs) and exploring the effect of carvedilol (a known anti-atherosclerosis drug) on BTMs, and comparing its probable effect to alendronate (a nitrogen-containing bisphosphonates used for treatment of osteoporosis).

The possible role of MMP9 in the pathogenesis of atherosclerosis in HFD fed rats through investigating the effect of induction of atherosclerosis on MMP-9 gene expression in aortic strips and the correlation of MMP-9 to dyslipidaemia and inflammatory markers, as well as its correlation to bone turnover markers, as a proposed link between atherosclerosis and osteoporosis.

## Materials and methods

### Experimental animals & groups

All animals’ procedures were performed in accordance to and approved by Cairo University Institutional Animal Care and Use Committee (CU- IACUC) [application number CU/III/F/61/17], and in accordance with the recommendations for the proper care and use of laboratory animals. Rats were anesthesized generally (Ketamine 80mg/kg and xylazine (10mg/kg), intraperitoneally) and were euthanized by decapitation.

This study was carried out in the animal house of National Cancer institute, Cairo University. 40 adult albino rats, 12 weeks of age, weights ranging from 150–200 gram were included in the study.

Rats were purchased and placed under ordinary living conditions in the animal house (temperature of 25±1°C; 12-hour light/dark cycle). They were housed in wire mesh cages in groups of 4 at their arrival and allowed to accommodate to their new environment for 1 week. All rats had free access to water and food. All animals’ procedures were performed in accordance to the Institutional Ethics Committee, Cairo University, and in accordance with the recommendations for the proper care and use of laboratory animals.

Animals were randomly divided into the following groups:

#### Group 1: Control group (n = 10)

Rats in this group were housed in standard cages (28 x 40 cm) with the same cage-mates and fed on standard laboratory rat chow (composition of standard rat chow: 5.4% fat, 53.8% carbohydrate, 21.9% protein, 2.9% fibre, mixture of minerals and vitamins obtained from animal house [12]) for the whole 8 weeks duration of the study.

Atherosclerosis was induced in the remaining rats by a combination of HFD (60% of total calories) + stress through chronic mild stress [13] and (unstable housing) [14] for 8 weeks [13] (a model of chronic mild stress (CMS) was induced by housing rats in crowded conditions composed of 4 rats per cage in a relatively small space (20x26cm). In addition, rats in the stress group were housed with a different combination of rats each day (unstable housing)).

The atherosclerosis -induced rats were subdivided into 3 groups each comprising 10 rats:

#### Group 2 (untreated atherosclerosis group) (n = 10)

Rats received daily vehicle of 0.5 ml saline by oral administration for 8 weeks.

#### Group 3 (CARV-treated atherosclerosis group) (n = 10)

Rats were treated with carvedilol (carvedilol (Coreg) drug was provided in the form of tablets from Sandoz Company, Tablets were dissolved in saline and administrated orally to rats, at a dose of (10mg/kg/d) [15]) (10 mg/kg) daily by oral gavage for 8 weeks [15].

#### Group 4 (alendronate treated atherosclerosis group) (n = 10)

Rats were treated with alendronate sodium (Fosamax (sodium alendronate): drug was provided in the form of tablets from Merck sharp & Dohme (MSD) Company, USA. Tablets were dissolved in saline and administrated orally to rats, at a dose of (3mg/kg/d)) (3 mg/kg) daily by oral gavage for 8 weeks [16].

### Experimental measurements

At the end of the 8 weeks experimental period, and after an overnight fast, blood samples were collected from retro-orbital plexus for estimation of Lipid profile [Total cholesterol, high density lipoproteins (HDL), triglycerides (TGs)], inflammatory markers (IL-6, TNF-α, CRP and NO) and Bone turnover markers (Alkaline phosphatase and osteocalcin as markers of bone formation and pyridinoline as marker of bone resorption).

Rats were then euthanized by decapitation, and the aortas and the tibias were carefully dissected. A part of each aortic and tibial tissue sample was frozen in liquid nitrogen and stored at -80C for estimation of Metalloproteinase 9 gene and protein expression, N-terminal propeptide of type I procollagen PINP, C-terminal telopeptide of type I collagen CTX and NF-kB. While the rest of aortic tissue samples were fixated in 10% formol saline for histopathological examination. Tibial samples were fixed in 4% neutral buffered formaldehyde, decalcified and processed for histopathological examination.

### Concise methodology

#### Biochemical measurements

*Estimation of serum cholesterol*. The total plasma cholesterol was measured by quantative–Enzymatic–Colorimetric determination of Total and HDL cholesterol in serum [17].

*Measurement of plasma Triglycerides*. The plasma Triglycerides was measured by quantative–Enzymatic–Colorimetric determination of Triglycerides in serum [18].

*Measurement of plasma HDL*. HDL- cholesterol is obtained through selective precipitation of LDL and VLDL lipoproteins, thus HDL lipoproteins remain in solution [19].

Estimation of serum TNF-α level through quantitative sandwich enzyme immunoassay technique [20].

Estimation of serum IL-6 level by colorimetry technique after employing an antibody specific for rat IL-6 [21].

Estimation of serum levels of alkaline phosphatase (ALP) through the quantitative sandwich enzyme immunoassay technique [22].

Estimation of serum levels of Osteocalcin (OC) through the competitive enzyme immunoassay technique [23].

Estimation of serum levels of pyridinoline through the Competitive-ELISA principle [24].

Estimation of CTX tissue levels by ELISA (LifeSpan BioSciences, Washington, United States) according to the manufacturer`s recommendations.

Estimation of PINP tissue levels by ELISA (MyBiosource, California, United States) according to the manufacturer`s recommendations.

Estimation of serum level of NO through the Double Antibody Sandwich Technique [25].

Estimation of MMP-9 protein expression in Aorta and tibia tissue samples using enzyme-linked immunosorbent assay kits (Elabscience Biotechnology Co Ltd, Beijing, China) according to the manufacturer’s instructions.

Detection of MMP-9 and NF-kB gene expression by real time Quantitative polymerase chain reaction (real time-PCR) in aorta and tibia of rats [26].

#### Quantitative real time PCR

*RNA extraction*. Total RNA was isolated using Qiagen tissue extraction kit (Qiagen, USA) according to instructions of manufacture.

*cDNA synthesis*. The total RNA (0.5–2μg) was used for cDNA conversion using high capacity cDNA reverse transcription kit Fermentas, USA).

*Real-time qPCR using SYBR Green I*. Real-time qPCR amplification and analysis were performed using an Applied Biosystem with software version 3.1 (StepOne™, USA). The qPCR assay with the primer sets were optimized at the annealing temperature. The primer sequence was shown in Table 1.

**Table 1:** The primer sequence of the studied genes.

	Primer sequence
MMP-9	Forward primer: 5-GTGGGAGAAAGTTTGCCAGG-3
	Reverse primer: 5- GTAGGAAGAGAGGGAAGAGG-3
NF.KB	Forward: 5’- GGT TCC CTG GCA TAA TCT GA -3
	Reverse: 5’- GTC ATC GAG ACC CCA AGG TA -3
Beta actin	Forward: 5′-ATCACCATCTTCCAGGAGCG -3′
	Reverse: 5′-CCTGCTTCACCACCTTCTTG-3′

Calculation of Relative Quantification (RQ) (relative expression): The relative quantitation was calculated according to Applied Bio system soft were using the following equation

∆ Ct = Ct gene test − Ct endogenous control

∆∆Ct = ∆Ct sample1 − ∆Ct calibrator

RQ = Relative quantification = 2-∆∆Ct

The RQ was the fold change compared to the calibrator (untreated sample).

#### Histological and morphometric examination

Specimens from aortae were obtained and fixated in 10% formol saline for 24 hours. Paraffin blocks were processed and serial transverse sections were obtained.

Serial 5 μm thick sections of aorta were subjected to haematoxylin & eosin (H&E) [27] and Masson’s trichrome stain [28]. In addition, Von Kossa staining for the aortic sections were performed to illustrate the vascular calcification.

*Morphometric studies*. Data were obtained using "Leica Qwin 500C" image analyser computer system Ltd. (Cambridge, England) in Faculty of Medicine, Cairo University. Each slide (10 slides / group) was examined using Olympus microscope and 10 non-overlapping randomly selected fields (10 fields/ slide) in magnification of (200x) were estimated. The following parameters were assessed:

- The thickness of the tunica media (μm) in H&E stained sections.
- The area % of collagen in Masson’s trichrome stained sections of aorta sections.

The midsection of the tibias were dissected out with sharp blade and fixed in 4% neutral buffered formaldehyde. Decalcification was performed in ethylene diaminetetra-acetic acid (EDTA) solution with PH 7 for about 4 weeks. The EDTA was refreshed every 3 days until a fine needle could easily be inserted into the bone. Decalcified specimens were then washed, dehydrated in gradient alcohol, embedded in paraffin wax. The decalcified specimens were processed for paraffin blocks and serial transverse sections from the diaphysis were obtained.

### Statistical analysis

Data were coded and entered using the statistical package SPSS version 25. Data was summarized using mean and standard deviation. Comparisons between groups were done using analysis of variance (ANOVA) with multiple comparisons post hoc test. Correlations between quantitative variables were done using Pearson correlation coefficient. P-values less than 0.05 were considered as statistically significant.

## Results

### Bone turn over markers (N-terminal propeptide of type I procollagen, Alkaline phosphatase, osteocalcin, C-terminal telopeptide of type I collagen, and pyridinoline)

The mean values of serum PINP, ALP, OC, CTX and pyridinoline in the untreated atherosclerotic group exhibited a significant increase as compared to their corresponding values in control, as shown in Table 2, which reflects the presence of a possible link between atherosclerosis and bone turnover markers.

**Table 2:** Comparison of the mean values of serum ALP, OC, and pyridinoline and tibial PINP and CTX among the studied groups.

	Control (n = 10)	Untreated Atherosclerosis group (n = 10)	CARV-treated Atherosclerosis group (n = 10)	Alendronate treated Atherosclerosis group (n = 10)
**ALP (IU/L)**	120.56±6.55	200.64±31.48*	159.28±22.27 #	155.04±14.94 #
**Osteocalcin (ng/ml)**	2.1±0.56	4.6±1.13 *	2.88±0.26 #	2.34±0.51 #
**Pyridinoline (nmol/L)**	1.6±0.5	3.14±0.42 *	1.83±0.53 #	2±0.51 #
**PINP (NG/MG PROTEIN)**	110.95±3.89	343.4±44.56*	180.23±19.56#	163.1±18.2#
**CTX (PG/MG PROTEIN)**	2.65±0.21	8.97±1.4*	4.08±1.2#	3.47±0.66#

Values are presented as mean ±SD

*: statistically significant as compared to corresponding value in the control group (P<0.05)

#: statistically significant as compared to corresponding value in the untreated Atherosclerosis group (P<0.05)

We noticed also a significant reduction in serum PINP, ALP, OC, CTX and pyridinoline in CARV treated group and in alendronate treated group when compared to the untreated atherosclerotic group, with no significant difference in all these parameters when compared to the control group. The two treated groups showed no significant difference in all these parameters as compared to each other, as shown in Table 2.

### Comparison of the morphological findings detected in the wall of dissected aorta between the studied groups

#### Haematoxylin and eosin results

**Fig 1:**
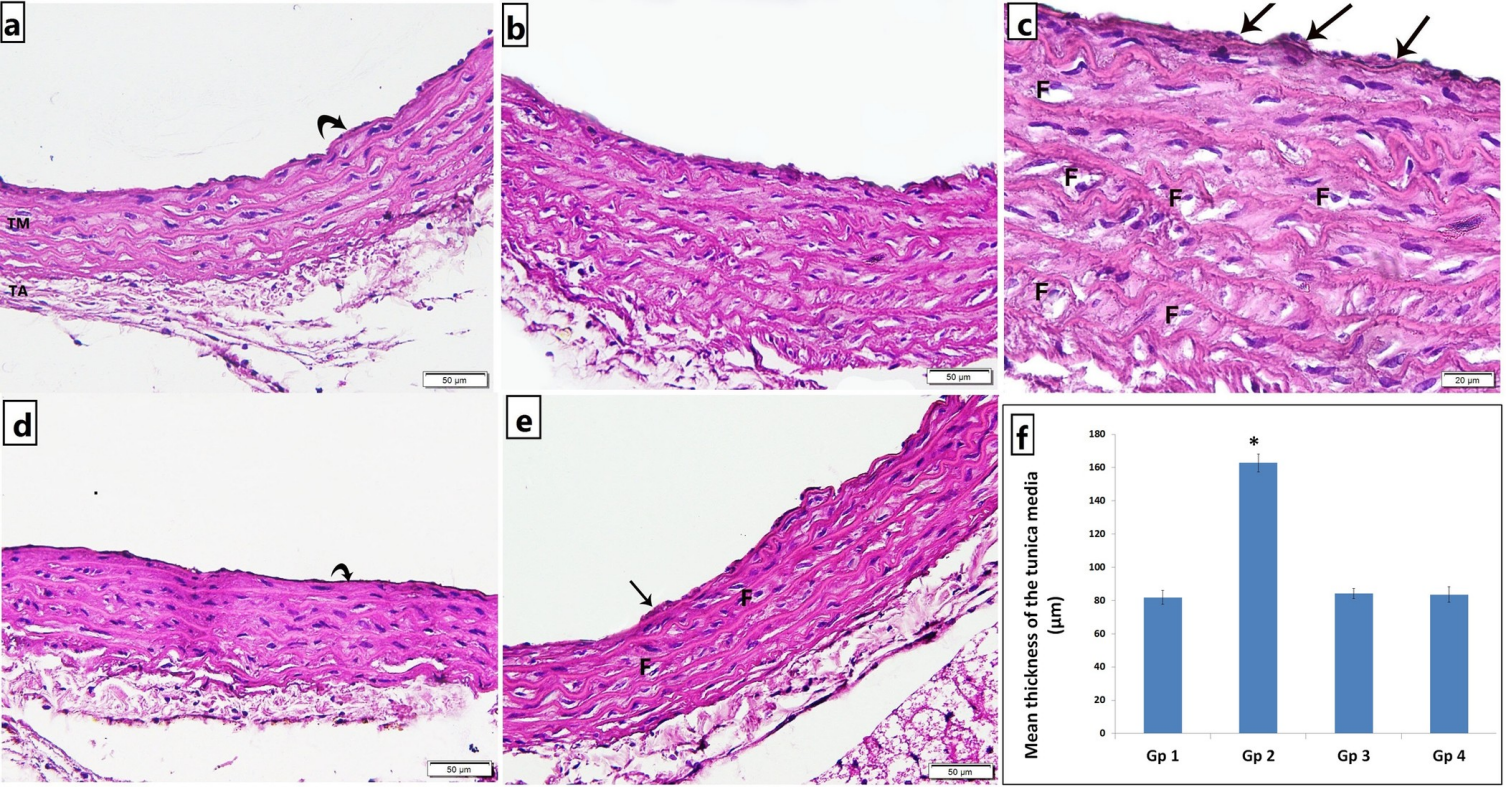
Photomicrograph of H&E stained abdominal aortic sections showing. a) Group 1 (control): normal structure of the three tunics; smooth tunica intima (curved arrow), tunica media (TM) and tunica adventitia (TA) with many continuous undulated elastic lamellae, b & c) Group 2 (untreated atherosclerosis): irregular endothelial surface, many focal areas of intimal thickening (arrows), numerous foam cells infiltration (F), increased thickness of tunica media with marked disorganization of smooth muscles and elastic lamellae, d) Group 3 (atherosclerosis+CARV): smooth tunica intima (curved arrow), properly arranged tunica media with continuous organized elastic lamellae., and e) Group 4 (atherosclerosis+alendronate): preserved nearly normal structure of the aorta with properly arranged elastic lamellae, yet a focal area of intimal thickening (arrow) and few foam cells (F) were noted [a,b,d,e x200; c x400]. f) Histogram illustrating the mean thickness of the tunica media. Values are presented as mean ±SD, *: statistically significant (P<0.05) compared to the corresponding value in Gp 1, Gp 1: control; Gp 2: untreated atherosclerosis; Gp 3: atherosclerosis+CARV; Gp 4: atherosclerosis+alendronate.

At the end of the 8 weeks period of our study, atherosclerosis was confirmed by histological examination of the aortic strips. The aortic tissue in the untreated atherosclerotic group showed rough irregular endothelial surfaces with many focal areas of thickening of the tunica intima, numerous foam cells infiltration, increased thickness of tunica media with marked proliferation of smooth muscles and disorganized elastic lamellae (Fig 1B and 1C). Treatment with CARV preserved nearly normal structure of aortic strips showing less foam cells, smooth tunica intima, properly arranged tunica media with continuous organized elastic lamellae (Fig 1D). Alendronate treatment in atherosclerotic rats preserved nearly normal structure of the aorta with properly arranged elastic lamellae, yet a focal area of intimal thickening and few foam cells were noted (Fig 1E). The mean thickness of the tunica media in the untreated atherosclerosis group was significantly higher as compared to the control group, yet, both carvedilol and alendronate treatment successfully restored it back to the normal range as in the control group (Fig 1F).

#### Masson’s Trichrome and Von Kossa stain results

**Fig 2:**
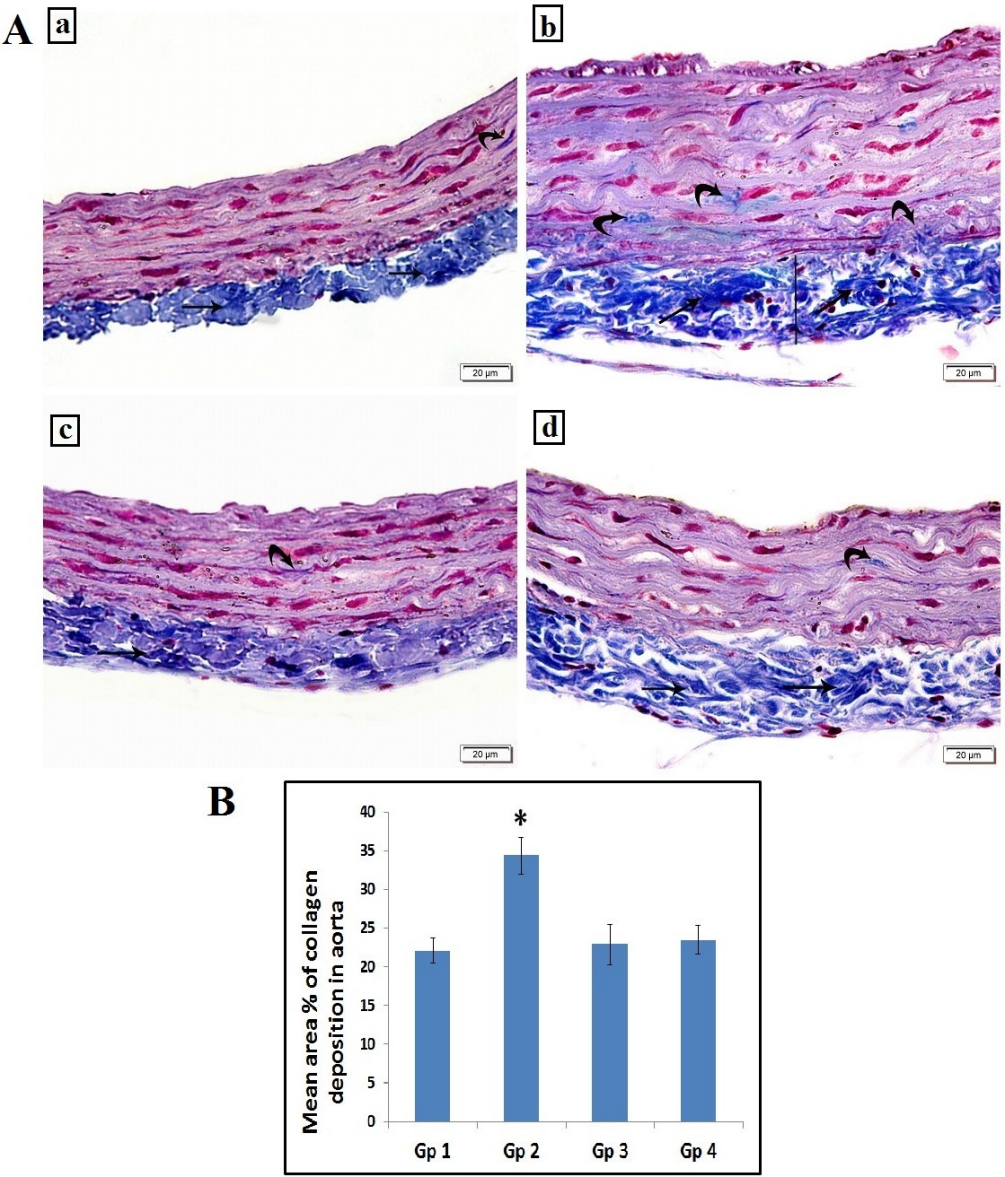
**A:** Photomicrograph of the abdominal aorta showing: (a) Group 1(control): normal collagen bundles distribution in tunica adventitia (arrow) with minimal collagen deposition in tunica media (curved arrow). (b) Group 2 (untreated atherosclerosis group): increased the thickness of tunica adventitia (line) with increased collagen deposition (arrows). Some collagen deposition is noted in tunica media (curved arrows). (c) Group 3 (atherosclerotic+ carvedilol): collagen bundles in tunica adventitia (arrow) with minimal collagen deposition in tunica media (curved arrow). (d) Group 4 (atherosclerotic+ alendronate): collagen bundles in tunica adventitia (arrow) with minimal collagen deposition in tunica media (curved arrow). Scale bar 20 μm (Masson’s Trichrome, x200). **B:** Histogram illustrating the mean area % of collagen deposition in aorta. Values are presented as mean ±SD, *: statistically significant compared to corresponding value in group 1 (P<0.05), Gp1: control group, Gp2: Untreated Atherosclerosis group, Gp3: atherosclerotic+ carvedilol, Gp4: atherosclerotic+ alendronate).

**Fig 3:**
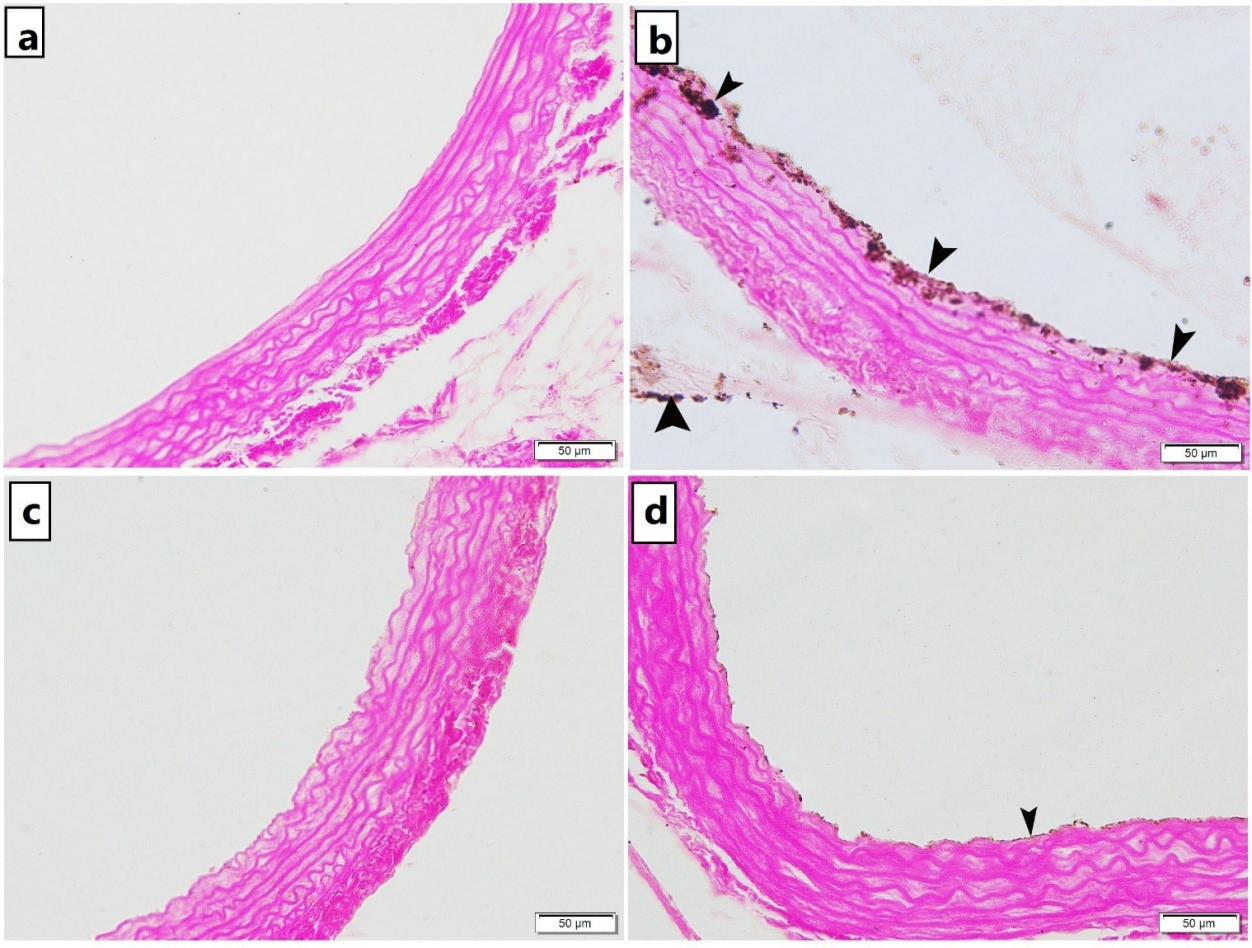
Photomicrograph of Von Kossa stained abdominal aortic sections showing. a) Group 1 (control): no calcium deposition were observed, b) Group 2 (untreated atherosclerosis): obvious black calcium particles deposition (arrowhead) in the tunica intima, tunica media and tunica adventitia c) Group 3 (CARV-treated atherosclerosis): and d) Group 4 (alendronate treated atherosclerosis): minimal scattered black calcium particles deposition in the tunica intima [x200].

Specimens from control rats displayed normal collagen distribution in the tunica adventitia with minimal collagen deposition in the tunica media (Fig 2A.a) and no calcifications (Fig 3A). Trichrome stained sections of untreated atherosclerotic group demonstrated an increase in thickness of the tunica adventitia with excessive collagen fibers deposition in the tunica media and the adventitia of the aorta (Fig 2A.b) and obvious black calcium particles deposition in the tunica intima, media and adventitia (Fig 3B). Atherosclerotic rats treated with carvedilol showed collagen bundles in the tunica adventitia with minimal collagen deposition in the tunica media (Fig 2A.c) and minimal scattered black calcium particles (Fig 3C). In alendronate treated atherosclerosis group, our results displayed a preserved normal distribution of collagen deposition in the tunica adventitia and the tunica media of the aorta (Fig 2A.d) and minimal scattered black calcium particles (Fig 3D). The mean area percentage% of collagen deposition in the untreated atherosclerosis group was significantly higher as compared to the control group, yet, both carvedilol and alendronate treatment successfully restored it back to the normal range as in the control group (Fig 2B).

### Gene and protein expression of MMP-9 in aortic strips and bone samples among all the studied groups

As shown in Table 3, induction of atherosclerosis by combination of HFD and stress for 8 weeks in untreated atherosclerotic group yielded a significant increase in the mean values of gene and protein expression of MMP_9 in extracted aortic and tibial tissues in this group as compared to the control group, thus denoting the dramatic effect of atherosclerosis induction on this molecule.

**Table 3:** Comparison of the mean values of gene and protein expression of MMP-9 among all the studied groups.

	Control (n = 10)	Untreated Atherosclerosis group (n = 10)	CARV-treated Atherosclerosis group (n = 10)	Alendronate treated Atherosclerosis group (n = 10)
**MMP_9 gene expression in vessel**	1.01±0.01	4.34±0.91 *	1.97±0.27 #	2.26±0.68 *#
**MMP-9 protein expression in vessel (ng/mg protein)**	73±12	218±7.3*	117±5.7*#	115±6*#
**MMP_9 gene expression in bone**	1±0.014	6.2±1.3*	2.9±0.31#	2.9±0.2#
**MMP-9 protein expression in bone (ng/mg protein)**	61±3.8	198±26*	92±13#	95±15#

Values are presented as mean ±SD

*: statistically significant compared to corresponding value in the control group (P<0.05)

#: statistically significant compared to corresponding value in the untreated atherosclerosis group (P<0.05)

Our results revealed a significant reduction in the mean values of MMP-9 tissues gene and protein expressions in CARV treated group and in alendronate treated group as compared to the untreated atherosclerotic group. The treated groups showed no significant variations in the mean values of MMP-9 as compared to each other (Table 3).

### Correlations between MMP-9 aortic gene expression and parameters of lipid profile, inflammation, NFKb, tibial MMP-9 and bone turnover parameters among the studied groups

To further emphasize the relation of MMP-9 gene expression with other parameters, correlation studies were done. On reviewing the results of correlation to identify factors most strongly correlated with MMP9 aortic gene expression, the present study revealed that the following parameters were significantly correlated with MMP9:

Serum levels of TC, TGs, HDL, serum TNF-α, IL6, CRP, nitric oxide, gene expression of NF-kB, tibial gene and protein expression of MMP9, serum ALP, OC and pyridinoline.

A significant positive correlation was noticed between MMP9 gene expression and serum TC and TGs (p <0.001, r = 0.845 and p <0.001, r = 0.796 respectively) while HDL showed a significant negative correlation with MMP-9 gene expression (p <0.001, r = -0.831).

A significant positive correlation was also noticed between MMP9 gene expression and TNF-α, IL6, CRP, nitric oxide and NF-kB gene expression (p<0.001, r = 0.827; p<0.001, r = 0.820; p<0.001, r = 0.889; p<0.001, r = 0.811 and p<0.001, r = 0.899 respectively).

MMP9 aortic gene expression was significantly positively correlated with serum ALP, OC, pyridinoline and MMP9 tibial gene and protein expression (p<0.001, r = 0.710; p<0.001, r = 0.749; p<0.001, r = 0.780; p<0.001, r = 0.897 and p<0.001, r = 0.879 respectively).

## Discussion

Obesity has been clearly associated with CVD, yet the relation between obesity and bone diseases is currently an intriguing area of research. A link between atherosclerosis and osteoporosis has been increasingly suggested through various biological and epidemiological studies, however, the precise molecular pathways underlying this close association remain poorly understood [29].

The present study added a strong evidence on this link through investigating the effect of induction of atherosclerosis, by combining high fat diet and stress, on bone turnover markers, exploring the effect of carvedilol, on bone turnover markers in atherosclerotic rats, and comparing its effect to alendronate effects.

At the end of the 8 weeks period of our study, atherosclerosis was confirmed by histological examination of the aortic strips. The aortic tissue of the untreated atherosclerotic group showed rough irregular endothelial surfaces with many focal areas of thickening of the tunica intima, numerous foam cells infiltration, increased thickness of tunica media with marked proliferation of smooth muscles and disorganized elastic lamellae in H&E staining. For more demonstration of atherosclerotic findings, Masson’s trichrome and Von Kossa staining were done and showed increased thickness of tunica adventitia with increased collagen deposition, with some collagen deposition noted in tunica media and obvious black calcium particles deposition in the tunica intima, media and adventitia.

Interestingly, estimation of bone turnover markers (BTMs) denoted a significant elevation of levels of both markers of bone formation, PINP, alkaline phosphatase (ALP) and osteocalcin (OC), and marker of bone resorption, CTX and pyridinoline, in the untreated atherosclerotic group as compared to the control group, in agreement with Idelevich et al. [30], and Hoshino et al. [31] results. Yet, the histopathological examination of tibial bone samples was not conclusive.

Several clinical studies illustrated the fracture predictive role of bone turnover markers (BTMs) in postmenopausal women independent of bone mineral density (BMD) [32, 33]. Increased bone turnover alters bone quality through altering bone microarchitecture. This microarchitectural alteration can be evaluated by BTMs while BMD can only measure the bone mass in fracture risk assessment [34].

Apart from their role in the skeletal system, BTMs play a role in atherosclerotic diseases. Analogous process features vascular calcification development and skeleton bone formation [35, 36]. ALP is shown to be involved in vascular calcification via the pyrophosphate pathway [37]. It also stimulates vascular smooth muscle cell trans-differentiation into chondrocyte-like cells [38], a pivotal step in vascular calcification. Moreover, the expression of OC in endothelial progenitor cells EPCs has been suggested as a marker of early disease in coronary atherosclerosis [39].

In our untreated atherosclerotic group, besides conveying an increased bone turnover, the increase in serum osteocalcin expression may reflect the osteogenic differentiation of VSMCs, and, the increase in serum pyridinoline may also reflect an increased degradation of extracellular matrix in atherosclerotic aorta denoting plaque instability.

While studying the effect of CARV on BTMs, our results revealed a significant reduction in levels of PINP, ALP, OC, CTX and pyridinoline in CARV treated group as compared to the untreated atherosclerotic group. To our knowledge, these results are the first to detect the effect of carvedilol treatment on bone turnover markers in atherosclerotic rat model.

Although CARV is not superior to traditional β-blockers in blood pressure control, it still shows great benefit in the inhibition of collagen deposition [40, 41].

Bones are innervated by sympathetic neurons, noradrenaline promotes bone resorption via β2-adrenergic receptors and also affects bone formation by inhibiting osteoblast proliferation [42]. Therefore, Carvedilol use may enhance bone formation and decrease fracture risk.

Intriguingly, the effect of alendronate on BTMs showed no significant difference as compared to CARV treated rats, and, on the flip side, alendronate effect on the atherosclerotic findings and fibrosis in the aortic strips showed no significant difference as compared to CARV treated rats, suggesting a common molecular pathway targeted by these drugs traditionally known to treat osteoporosis and atherosclerosis respectively.

With the increasing interest in ECM and MMPs role in tissue remodelling and disease development, the current study explored the role of MMP-9 in the pathogenesis of atherosclerosis through investigating the effect of induction of atherosclerosis on MMP-9 gene expression in aortic strips and its correlation to atherosclerosis developing factors (dyslipidaemia and inflammatory markers). We also explored its correlation to bone turnover markers in order to illustrate MMP-9 as a probable molecular link tying the pathogenesis of atherosclerosis and osteoporosis.

MMP-9 gene and protein expression in the current study was significantly higher in the aortic strips of the untreated atherosclerotic rats as compared to the control group which supports Konstantino et al. [43], while opposes Cavusoglu et al. [44], results. And, When Pearson correlation was performed among all studied groups, our results revealed a significant correlation between tissue expression of MMP-9 and markers of dyslipidaemia, in agreement with Ardans et al. [45]. There was also a significant correlation between aortic MMP-9 and markers of inflammation as well as tibial MMP9 and bone turnover markers.

Whilst previous studies found that MMP9 levels were significantly associated with increased bone resorption in osteoporosis [10], others identified MMP-9 as a strong independent predictor of atherosclerotic plaque instability in stable coronary heart disease patients, its level is directly associated with the size of the necrotic core of the coronary atherosclerotic plaque [46].

Among the macrophage phenotypes, foam cells are the leading source of active MMP-9 [47]. One proposed role of MMP-9 in atherosclerosis is promoting the migration of vascular smooth muscle cells through the internal elastic lamina. Moreover, an excess of MMP9 activity contributes significantly to ECM collagen destruction rendering the plaque more prone to rupture [48].

The significant correlation observed between MMP-9 expression and serum markers of dyslipidaemia may be attributed to the fact that dyslipidaemia results in increased levels of circulating monocytes and neutrophils, sources of MMP-9, through increasing proliferation and mobilization of innate immune cells, that foster inflammation [49]. This assumption is further supported by our results that showed a significant positive correlation between MMP9 tissue expression and inflammatory markers among all studied groups.

Recent attention has been focused on the potential role of MMP-9 as a circulating biomarker of inflammation [50]. Indeed, a strong cross talk between MMP9 and inflammatory response exists.

Previous studies showed the role of TNF-α in upregulation of MMP expression in vascular endothelial cells [51], activation of its production [52], and its secretion [53]. Besides, IL-6 induces macrophage expression of MMP-9 [54]. And, local CRP levels might probably uphold OxLDL uptake and MMP-9 induction by macrophages [55].

Furthermore, previous studies on hCRP-treated rats have displayed an increased NF-κB DNA binding activity in the nuclear extract of pouch macrophages. So, in vivo CRP activation of the NF-κB pathway is appealing. NF-κB sounds as an upstream molecule in CRP-mediated MMP-9 induction [56, 57]. In fact, NF-kB is needed for cytokine up-regulation of MMP 9 in VSMCs [58].

Our results also noticed a positive correlation between MMP9 expression and NO level which is supported by Sigala et al. [59], who noticed that both MMP-9 and iNOS expression levels were higher in atherosclerotic patients.

As regards the association between MMP9 expression and bone turnover markers, our results discerned a positive correlation between MMP9 and ALP, osteocalcin and pyridinoline in agreement with Christian et al. [60], Gluba-Brzózka et al. [61], and Martos et al. [62], respectively.

To conclude, the matrix metalloproteinase (MMP-9), responsible for degradation of collagen fibres, has been shown in the present work to be overexpressed in the aortic strips of atherosclerosis model. More importantly, its tissue expression correlated with atherosclerosis risk factors (dyslipidaemia and inflammatory markers) and with bone turnover markers, and hence it may be a key molecule in charge for coupling these two tissue destruction pathologies.

The present study’s results are clearly dissolving doubts about the pathogenic association of atherosclerosis and osteoporosis in high fat diet fed rats.

This has boosted our interest in identifying pharmacologic agents that through targeting MMP-9 can provide benefits in terms of slowing the progression of atherosclerosis and also can protect against deterioration of bone quality.

Expression of MMP-9 has been described in osteoclast cells [63], and also in macrophages, SMCs and endothelial cells derived from atherosclerotic plaque material [48]. Macrophages and osteoclasts are highly endocytic cells that share the same lineage. The protective effect of alendronate on macrophages and on osteoclasts in vitro are astonishingly the same. Hence, both cells have ability to internalise bisphosphonates, therefore, both are ideal targets for these drugs [64].

Interestingly, our work revealed that alendronate treatment significantly reduced tissue level of MMP9 gene and protein expression in alendronate treated atherosclerotic rats when compared with the untreated group, in agreement with Melani et al. [65], results.

Alendronate stops the macrophage lineage development in mouse bone marrow cultures, counteracts the macrophages bone particles breakdown, and trigger apoptosis of mouse macrophage cell lines. With increased apoptosis of macrophages, alendronate causes subsequent reduction in MMP9 expression as these two cells are the main cells producing MMP9 [64].

It is to be noticed that no significant variations in the mean values of all studied parameters between CARV-treated group and alendronate treated group.

Concerning CARV, our results revealed a significant decrease in MMP9 expression in aortic strips of CARV-atherosclerotic rats group compared to their untreated counterpart. The reducing effect of CARV on MMP9 levels is probably indirect and may be related to TNF-α [66].

These results are in agreement with Wu et al. [67], who noticed that CARV treatment inhibited MMP9 expression in atherosclerotic rat model and Raimundo Fernandes et al. [68], who demonstrated a reduction in the formation of MMP-9 with carvedilol treatment in an experimental model of periodontal disease.

## Conclusions

MMP_9 is a pivotal molecule that impact the atherogenic environment of the vessel wall. A strong cross talk exists between MMP-9, cytokine production and macrophage function. It also plays an important regulatory role in osteoclastogenesis. So, it may be a key molecule in charge for coupling CVD and bone diseases in high fat diet fed rats. Therefore, we hypothesized that MMP-9 is a worthy molecule to be targeted pharmacologically in order to control both conditions simultaneously. Our results has supported our hypothesis, and we believe that this study represents an important addition that has shed light on an important research gap and unrevealed for the first time MMP9 as a possible molecular link tying atherosclerosis and osteoporosis, which can be blotted further in different studies to further investigate the specific mechanisms involved and to further invest and to translate this hypothesis into clinical studies and guidelines.

## Supporting information

S1 File (DOCX)
