# Supplementary material for: Matrix metalloproteinase 9 a potential major player connecting atherosclerosis and osteoporosis in high fat diet fed rats
Source: PLoS One. 2021 Feb 11;16(2):e0244650. doi: 10.1371/journal.pone.0244650 (PMC7877768; doi:10.1371/journal.pone.0244650)
Supplement: S1 File — (DOCX) [file pone.0244650.s001.docx]

# **Supplemental data:**

## **Lipid profile: (Serum cholesterol, TGs, HDL):**

As shown in table (4), the effect of combination of HFD and stress for 8 weeks on untreated atherosclerotic resulted in marked significant increase in serum cholesterol and TGs with a significant reduction in HDL with mean value compared with the control group.

Also, the effect of CARV treatment in group 3 yielded a significant decrease ((P-value <0.05) in the mean values of cholesterol and a significant elevation in HDL and alendronate treated group in group 4 showed a significant decrease in the mean values of cholesterol and TGs with a significant increase in HDL level when compared with untreated atherosclerotic group, however, no significant changes in the level of these markers were observed between the two treated groups and both drugs cannot return these markers back to the control group values as noticed in the same table.

**Table (4): Comparison of the mean values of serum cholesterol, TGs and HDL among all studied groups**

|  | **Control** | **Untreated Atherosclerosis group** | **CARV-treated Atherosclerosis group** | **Alendronate treated Atherosclerosis group** |
| --- | --- | --- | --- | --- |
| **Cholesterol(mg/dl)** | 136±12.04 | 250.4±39.76 * | 183.4±12.62 *# | 177.8±11.03 # |
| **TGs (mg/dl)** | 68±8.75 | 111.6±12.46 * | 90.4±5.13 *# | 86.2±9.6 *# |
| **HDL (mg/dl)** | 57.6±3.3 | 23.62±3.95 * | 42.56±5.22 *# | 43.26±6.49 *# |

*Values are presented as mean ±SD*: statistically significant compared to corresponding value in group (1A) (P<0.05)*

*#: statistically significant compared to corresponding value in group (2A) (P<0.05)*

*$: statistically significant compared to corresponding value in group (2B) (P<0.05)*

## **Inflammatory indices: (TNF, IL-6, CRP, NF-KB and Nitric oxide):**

As shown in table (5) , our results reported a highly significant elevation in the mean values of serum TNF-α , IL-6 , hsCRP, NO and NF_KB gene expression in aortic strips in response to combination of HFD + stress for 8 weeks in the untreated atherosclerotic group when compared with corresponding values of control group.

We noticed a remarkable significant reduction in the mean values of serum levels of TNF-α, IL-6, hsCRP, NO and NF_KB gene expression in aortic strips in CARV treated group denoting its anti-inflammatory role and we also noticed an obvious significant reduction in the mean values of these markers in alendronate treated group when compared with untreated atherosclerotic group, but no significant changes was detected in these parameters between the two treated groups , also the drugs cannot return these markers to the control values as noticed in table (3).

**Table (5): Comparison of the mean values of serum TNF-α, IL-6, hsCR and Nitric oxide among all the studied groups**

|  | **Control** | **Untreated Atherosclerosis group** | **CARV-treated Atherosclerosis group** | **Alendronate treated Atherosclerosis group** |
| --- | --- | --- | --- | --- |
| **TNF-α (ng/ml)** | 16.36±3 | 82.66±10.75 * | 38.38±11.88 *# | 37.36±10.72 *# |
| **NF_KB gene expression** | 1.01±0.03 | 5.18±0.69 * | 2.2±0.78 *# | 2.37±0.5 *# |
| **CRP(ng/ml)** | 0.89±0.15 | 5.24±1.8 * | 1.98±0.54 # | 1.88±0.3 # |
| **NO**  **(µmol/l** ) | 13.6±2.83 | 76.2±16.14 * | 37.82±9.47 # | 34.24±13.26 # |

*Values are presented as mean ±SD*

**: statistically significant compared to corresponding value in group ( 1A)(P<0.05)*

*#: statistically significant compared to corresponding value in group (2A)(P<0.05)*

*$: statistically significant compared to corresponding value in group (2B) (P<0.05)*
